# Multinutrient Biofortification of Maize (*Zea mays* L.) in Africa: Current Status, Opportunities and Limitations

**DOI:** 10.3390/nu13031039

**Published:** 2021-03-23

**Authors:** Nakai Goredema-Matongera, Thokozile Ndhlela, Cosmos Magorokosho, Casper N. Kamutando, Angeline van Biljon, Maryke Labuschagne

**Affiliations:** 1 Maize Breeding Program, Crop Breeding Institute, Causeway, Harare 00263, Zimbabwe; nakaigoredema@gmail.com; 2 International Maize and Wheat Improvement Centre (CIMMYT), Mount Pleasant, Harare 00263, Zimbabwe; t.ndhlela@cgiar.org (T.N.); c.magorokosho@cgiar.org (C.M.); 3 Department of Plant Sciences, University of the Free State, Bloemfontein 9300, South Africa; avbiljon@ufs.ac.za; 4 Department of Plant Production Sciences and Technologies, University of Zimbabwe, Mount Pleasant, Harare 00263, Zimbabwe; kamutandocn@gmail.com

**Keywords:** biofortification, multinutrient maize, provitamin A, quality protein maize, zinc

## Abstract

Macro and micronutrient deficiencies pose serious health challenges globally, with the largest impact in developing regions such as subSaharan Africa (SSA), Latin America and South Asia. Maize is a good source of calories but contains low concentrations of essential nutrients. Major limiting nutrients in maize-based diets are essential amino acids such as lysine and tryptophan, and micronutrients such as vitamin A, zinc (Zn) and iron (Fe). Responding to these challenges, separate maize biofortification programs have been designed worldwide, resulting in several cultivars with high levels of provitamin A, lysine, tryptophan, Zn and Fe being commercialized. This strategy of developing single-nutrient biofortified cultivars does not address the nutrient deficiency challenges in SSA in an integrated manner. Hence, development of maize with multinutritional attributes can be a sustainable and cost-effective strategy for addressing the problem of nutrient deficiencies in SSA. This review provides a synopsis of the health challenges associated with Zn, provitamin A and tryptophan deficiencies and link these to vulnerable societies; a synthesis of past and present intervention measures for addressing nutrient deficiencies in SSA; and a discussion on the possibility of developing maize with multinutritional quality attributes, but also with adaptation to stress conditions in SSA.

## 1. Introduction

Deficiencies of essential macro and micronutrients in human diets pose serious health challenges worldwide [1,2], although the impacts are greatest in developing countries [3,4]. Diets in developing regions such as subSaharan Africa (SSA) are characterized by insufficient quantities of multiple nutrients since the majority of people heavily depend on cereals such as maize and cassava [5,6]. Maize, in particular, is very rich in carbohydrates but very limited in other nutrients such as the essential amino acids lysine and tryptophan [7], and vitamin A [8]. The maize endosperm has low quantities of important minerals, such as Fe and Zn [9,10].

Because of over-reliance on maize in SSA, diseases induced by nutrient deficiencies such as kwashiorkor and pellagra that are caused by lack of proteins and tryptophan, respectively [7]; night blindness as a result of lack of vitamin A [11]; and acute respiratory infections induced by Zn deficiency [12], are common. These forms of malnutrition are also observed in many developed nations, although prevalence is less because advanced economies allow people to diversify their diets with highly nutritious foods [13]. For instance, balancing diets with protein sources such as beans, peas, fish, meat and milk [14], fruits and vegetables rich in vitamins [15], and carbohydrates from cereals [6], can inhibit malnutrition challenges, but most families in developing countries cannot afford balanced diets [3]. Therefore, effective intervention strategies are required to save lives in developing countries. Such interventions include crop biofortification, advocated as the most appropriate, cost-effective and sustainable intervention that has widespread coverage in minimizing nutrient deficiencies globally [10,16].

The term biofortification, in general, refers to the biological enrichment of food crops with either macro or micronutrients by means of agronomic practices, conventional plant breeding or genetic engineering [17]. Through conventional and molecular-based breeding techniques, several biofortified maize cultivars have been commercialized in SSA [18], but biofortification was designed solely for specific limiting nutrients, either Zn, provitamin A or lysine or tryptophan [7,19,20]. These cultivars, however, cannot fully meet the challenges on the ground, because the macro and micronutrient deficiencies in SSA are complex and cannot be addressed by one nutrient; and maize production in this region is done by small-scale farmers who rarely diversify cultivars with different nutritional attributes; hence growing a provitamin A cultivar does not help the farmers to address Zn or lysine and tryptophan deficiencies.

Because of these reasons, it is important to look at possibilities of stacking nutritional quality traits in a single cultivar. During the breeding process, breeders should also pay attention to the production environments to which biofortified cultivars are adapted. For instance, maize production in SSA is constrained by abiotic stresses such as heat, drought and low soil nitrogen [21,22], as well as biotic stresses including diseases and insect pests [23]. Therefore, resistance or tolerance of multinutrient maize cultivars to these stress conditions will contribute to food and nutritional security in the most vulnerable populations. This review focuses on providing a synopsis of the health challenges associated with macro and micronutrient deficiencies and links these to societies that are most at risk. A synthesis of past and present intervention measures to addressing nutrient deficiencies in SSA is considered and the possibility of developing maize with multinutritional quality attributes, but adapted to stress conditions in SSA, is discussed.

## 2. Maize Uses, Malnutrition Prevalence and Health Risks of Maize-Based Diets in SSA

### 2.1. Maize Is “Life” in Africa

Food inequalities still exist even in the 21st century, where some people in the world subsist on monotonous, cereal-based diets. For instance, in SSA maize alone provides more than 30% of total calories in more than 20 countries [18,24]. Because s it serves as a staple food for most African countries, daily per capita consumption in SSA exceeds 330 g, providing protein and energy [20,25]. In addition to calories, maize is a source of micronutrients and phytochemicals, such as anthocyanins, carotenoids and phenolics, as well as dietary fiber, and these are vital for disease prevention [18].

Different maize food products are consumed across the world, and in African countries maize meal is commonly prepared from dried grain, and sometimes consumed as boiled or roasted green maize [25]. In this way, maize is used to bridge the long dry “hunger-season” before harvesting, ensuring food security for many people [26]. Apart from its use as human food, maize contributes significantly to the livestock-to-meat cycle across the world and has various industrial purposes, including ethanol and biofuel production [20,27]. High per capita consumption of conventional maize, coupled with limited supplementary food sources, exposes African societies to a greater risk of protein, vitamin A and deficiencies of minerals such as Zn and Fe [28,29].

### 2.2. Prevalence and Impact of Zn, Provitamin A and Protein Deficiency in SSA

**Figure 1:**
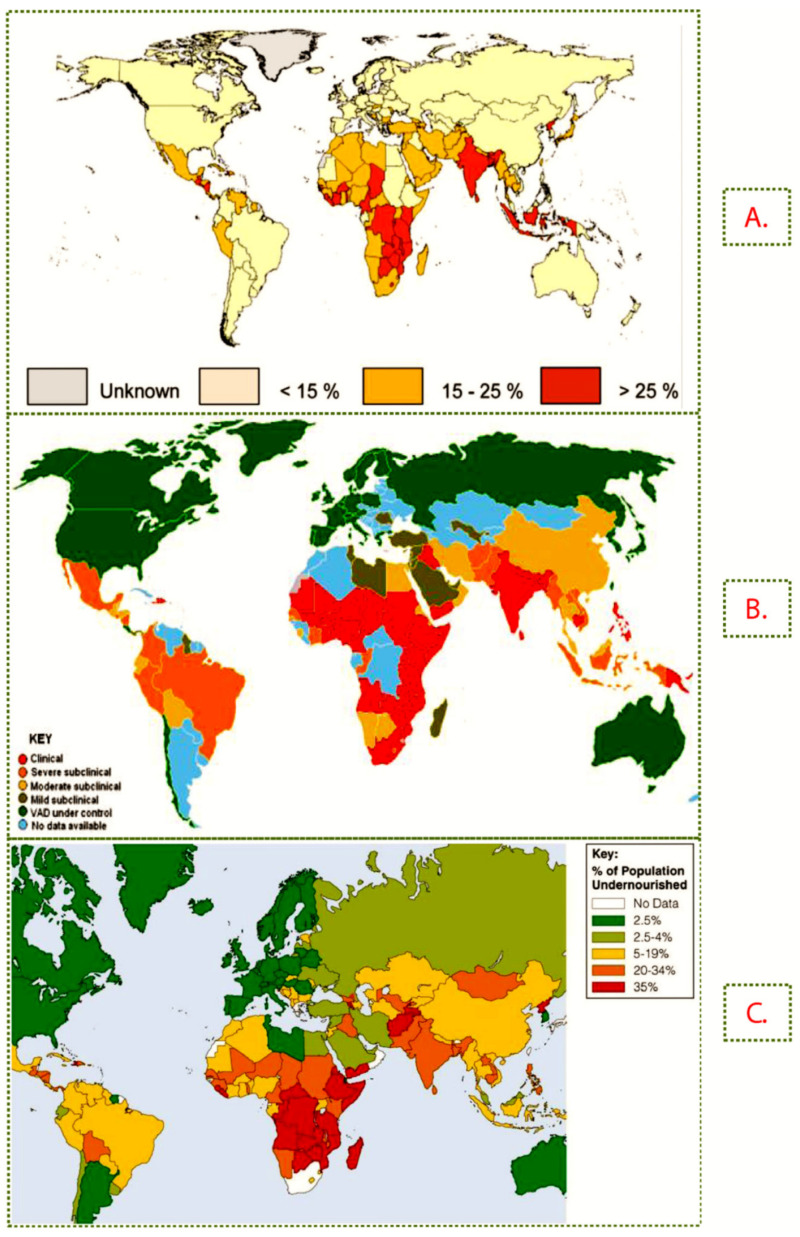
The estimated prevalence of (**A**) Zn deficiency (**B**) vitamin A deficiency (**C**) protein deficiency across the world [4,37].

Statistics show that the global burden of both macro and micronutrient deficiency rose by more than 50% in the period between 1990 and 2010 [30]. Figure 1 shows the prevalence of Zn, vitamin A and protein deficiency in the world. Zn deficiency affects approximately one-third of the world population as a result of inadequate dietary Zn intake [31], and more than 25% of the affected people are from SSA [3,4]. Zn and vitamin A deficiencies are among the 10 top leading risk factor causes of Disability Adjusted Life Years (DALYs) in low-income countries (Table 1) [32]. In comparison to other micronutrients, vitamin A deficiency accounts for the greatest disease burden and infant mortality in developing countries. Vitamin A deficiency prevalence in Africa is estimated at approximately 48%, indicating a linear increase from the 40% reported in 1991 [33]. The WHO estimates that 7.8% of pregnant women in Africa have low serum retinol concentrations [34]. The prevalence of eye problems due to vitamin A deficiency in preschool children in this region stands at about 26.0% of all cases across the world [11].

In as much as Zn and vitamin A are important, the improvement of protein quality has long been a priority, dating back to the 1950s when the main focus was to alleviate protein-energy malnutrition prevalent in developing countries [35]. However, protein deficiency has remained a leading health risk factor in developing countries and affects about 54% of the preschool children in this region [36]. All these statistics indicate that societies in SSA, among other low-medium income countries, are at greater risk of malnutrition than in developed nations.

### 2.3. Dietary Reference Intake

Dietary Reference Intakes (DRIs) is a generic term used for nutrient reference values, including Recommended Dietary Allowance (RDA) and Estimated Average Requirement (EAR). It defines the lowest continuing intake level of a nutrient that is required to maintain a target level of nutrition in an individual [7]. DRI of vitamin A is usually expressed as retinol activity equivalent (RAE). In that regard, the recommended DRI for vitamin A for expectant mothers is 770 RAE, whereas for men and nonpregnant women it is about 700 RAE [37]. Similarly, the estimated average requirement of all adults for Zn is 1860 µg/day [1,38]. For the amino acids lysine and tryptophan, the DRI is approximately 22 and 6 mg/kg^−1^ per day respectively [39]. Nutrient requirements may vary with gender, age and whether women are lactating or not [40]. DRIs are used by plant breeders, geneticists and nutritionists to define breeding targets for different nutrients.

### 2.4. Physiological Functions of Vitamin A, Zinc, Lysine and Tryptophan

Vitamin A, Zn and essential amino acids such as lysine and tryptophan are important nutrients for growth and development in humans. Lysine and tryptophan are building blocks of proteins, whereas vitamin A and Zn are required in minute quantities but play crucial roles in metabolism [41]. For instance, low serum retinol in the diet causes night blindness, maternal deaths in pregnant women [42], and also increases infant morbidity and mortality rates during the first year of life [11,36]. On the other hand, Zn is described as a “metal of life” because it is a component of more than 300 enzymes in various types of body tissues [43,44]. It prevents organ inflammation and damage by reducing oxidative stress and functions in the regulation of blood pressure [45].

Protein, Zn and vitamin A malnutrition negatively affect cognitive development, reduces the ability to do physical work, and increase susceptibility to acute respiratory infections, diarrhea and a weakened immune system [29,31,34]. Lack of dietary protein in general, adversely affects the overall well-being of infants, and symptoms that include peripheral oedema, diarrhea and severe wasting, are collectively known as “kwashiorkor” [35]. Lack of tryptophan, a precursor amino acid for niacin (vitamin B3) synthesis causes pellagra in children [46]. Symptoms of pellagra include diarrhea, inflamed skin, dementia and sores in the mouth [47]. Pellagra usually affects children between six months and four years who are breastfed by malnourished mothers and weaned on maize-based foods without any supplementation. Several studies report that malnutrition as a result of inadequate intake of essential amino acids and micronutrients such as Zn and vitamin A can be more devastating than low intake of carbohydrates, because essential amino acids are required in most metabolic processes [12,48]. Both protein and micronutrient deficiency cause irreversible life-threatening health consequences, including permanent damage of important body organs such as the brain, liver, kidneys, and the endocrine and central nervous systems [49,50]. In an effort to alleviate these adverse health effects of nutrient deficiency, several interventions have been proposed.

## 3. Strategies to Alleviate Vitamin A, Zinc and Protein Malnutrition

Several interventions have been used to increase dietary intake of nutrients. These include industrial fortification [51], clinical or pharmaceutical supplementation, dietary diversification [12] and crop biofortification [1,52]. Crop biofortification is further classified as agronomic and genetic biofortification [5,53]. It is important to note that all these interventions are only complementary, implying that it is better to use them in an integrated manner than as mutually exclusive. The choice of these strategies is highly influenced by several factors including resources, accessibility, affordability, sustainability and technical feasibility. Based on these parameters, genetic biofortification would be the most appropriate, cost-effective and sustainable intervention in minimizing nutrient deficiencies in communities subsisting on maize-based diets in Africa [10,16]. The advantages and disadvantages of each of these interventions are discussed in the following sub-sections.

### 3.1. Industrial Fortification

Enriching food with essential nutrients such as minerals and vitamins either comes as a commercial choice by food processors or a stipulated government policy to curb certain nutritional deficiencies [40]. In SSA, population-based food fortification programs, including large-scale fortification of staple foods, have been widely used. For instance, South Africa, Zimbabwe, Nigeria, Uganda, Malawi and Kenya have embraced food fortification initiatives from their respective governments. In these countries, basic commodities such as salt, bread, maize meal, wheat flour, sugar, cooking oil and infant formulas are fortified with a wide range of vitamins including vitamin A and nicotinamide, and minerals such as iodine, Zn and Fe [54]. Industrial fortification of table salt in Africa dates back to 1990s, when it was fortified with iodine to prevent goiter [55]. This initiative was successful due to support from the government, and iodization of table salt became compulsory. Fortification of maize-based foods with essential amino acids lysine, tryptophan and methionine has been reported in Nigeria to improve food protein value [56].

Despite its wide coverage in curbing nutrient deficiencies, food fortification has a number of limitations. It can cause health hazards due to toxicity, especially when food processors exceed the stipulated dose [57] due to lack of efficient quality monitoring systems, and hence may expose consumers to mineral toxicity [40]. Another disadvantage of this intervention is that costs incurred by the food processors are included in the price of a commodity and, consequently, the consumer has to bear all these costs [51]. As a result, fortified foods become more expensive than nonfortified foods. To cut production costs, food processors can default on the stipulated statutory requirements. Findings of a survey conducted in South Africa on fortified wheat and maize meal revealed that food processors add insufficient micronutrients as a cost cutting measure [58]. The difference in taste of fortified foods can reduce their acceptability by consumers. Relying on fortified foods to curb various nutrient deficiencies has low rural coverage, since the majority of rural people subsist on home-based food products [51,59].

### 3.2. Pharmaceutical Supplementation

Dietary supplements could be a useful intervention to mitigate the effects of various forms of nutrient deficiency in targeted communities [60]. Dietary supplements can be purchased in pharmacies or given as supplementation programs initiated by the government or donor-funded organizations [61]. Dietary Zn supplements include Zn sulphate (ZnSO_4_), Zn acetate, Zn gluconate and Zn oxide (ZnO) and Zn amino acid chelates [61,62]. The recommended dose for Zn supplements may differ with age, with infants below 36 months given 5 mg/day and dosage increases to 10 mg/day as the child grows [40]. Vitamin supplements occur in various forms, targeted to address a specific vitamin such as vitamin A and C, or as multivitamins. In addition to pharmacies, children born in SSA are immunized from 6–59 months old as part of postnatal care [28]. Protein, creatine and amino acid supplements are also widely marketed in high income countries to supplement diets for habitually active consumers, athletes and gym-goers [63]. Because of affordability issues, the use of such protein supplements may be irrelevant in alleviating protein malnutrition in SSA.

Success stories for the use of dietary supplements have been reported across the world, including SSA. For instance, the administration of dietary Zn supplements has been reported to increase the linear growth patterns and weight gain in children, and corrected hormonal imbalances in adolescents, in low-income countries [60,64]. Zn supplements have been useful for prevention of pneumonia in children under the age of five [65], and organ damage in diabetic patients [66,67]. Similarly, vitamin A supplementation has been widely adopted by several countries in Africa as government initiatives or as support to programs funded by nongovernmental organizations [68].

Despite all the health benefits of dietary supplements, this intervention has been associated with several limitations. Toxicity from overdose of dietary supplements may arise. Zn and vitamin A toxicity were reported to cause severe abdominal pains, nausea and vomiting in some patients [61]. In addition, chronic retinoid toxicity was reported to cause hyperpigmentation, dermatitis and irreversible renal dysfunction [69]. The unavailability and limited access to pharmacies or clinics, which is a common phenomenon in most developing countries, is a huge barrier for both effective and sustainable use of this intervention [70]. In addition, most developing countries rely on imports and donor-funded organizations for many kinds of medicines, including dietary supplements. Therefore, supply and demand of these dietary supplements could be affected by socio-economic or political instability that occur periodically [13]. In politically stable countries that depend on nongovernmental organizations for dietary supplements, projects may cease, or the donors may change their priorities. Compliance to dietary supplements is a key determinant for success of this intervention to alleviate malnutrition. Because compliance depends on the literacy level, limited compliance is common in some of the remote SSA communities [71]. Furthermore, dietary supplements are expensive for the majority of people in SSA [70], and therefore sustainable alternatives matching local population norms are needed.

### 3.3. Dietary Diversification

Dietary diversification is an intervention to change household diets to increase the variety and quantity of micronutrient-rich foods and animal-based food sources [72]. People who diversify their foods are at less risk of macro and micronutrient deficiency. This strategy is practical and feasible as a long-term solution [64]. Animal-based foods such as red meat, eggs, cheese, fish and seafood have relatively high Zn contents, where 100 g can provide up to 40% of the recommended daily Zn intake [73]. In addition to Zn, meat, fish and seafood contain bioavailable calcium, Fe, iodine, vitamin A, essential fatty acids and amino acids, including lysine and tryptophan [74]. It has been reported that 100 g of whole fish contribute significantly to the RDA for several nutrients, although nutrient composition varies with fish type, habitat and season [74]. Plant-based foods such as chickpeas, lentils and beans also contain substantial amounts of Zn and essential amino acids [75]. Provitamin A-rich fruits and vegetables include butternuts, carrots, mangoes and papayas, whereas liver, milk, and eggs are some of the animal-based sources of vitamin A. Although the consumption of nutrient-dense foods increases the chance of consuming adequate quantities of quality protein, vitamin A and Zn, the limitation of this food-based approach is affordability for the majority of people in low-medium income countries [76]. Furthermore, some vitamin A-dense fruits and vegetables such as papayas, butternuts and mangoes are seasonal, and are sometimes unavailable in the market.

Whilst consumption of diversified foods is unaffordable for a large fraction of the population, reduced nutrient bioavailability in some foods prohibits maximum absorption [77]. For instance, absorption of minerals such as Zn and Fe is largely affected by the presence of phytic acid (inositol hexakisphosphate), which chelates minerals, and reduces bioavailability. Other factors that may influence nutrient bioavailability include the food matrix, food preparation techniques, gut integrity and nutrient interactions [75]. Reduced bioavailability of β-carotene has been reported in foods with complex matrices such as maize and dark green leafy vegetables, and higher in foods with simpler food matrices such as fruit and red palm oil [78]. To increase bioavailability of these carotenoids, co-consumption with foods that contain monounsaturated fatty acids, such as canola and sunflower oil, is recommended [79]. A constant supply of diversified foods for poor communities can be achieved through growing a wide range of highly nutritious crops in nutri-farms [80]. This, however, requires technical support from governments through agricultural extension services to maintain these diversified nutri-farms, otherwise people may resort to crops of their own choice.

### 3.4. Agronomic Practices

Agronomic practices to improve Zn content in maize kernels has been widely reported through application of Zn fertilizers such as ZnO, Zn-EDTA and ZnSO4 [10,81]. This strategy could work for countries with Zn-rich soils but may be of little benefit to most countries in SSA because of low inherent Zn content, ranging from 5–55 mg/kg. Any application of Zn fertilizers may benefit the crop by increasing its yield, but without partitioning the much-needed micronutrient to the grain. In addition, the price of Zn fertilizers in SSA is a huge barrier to the use of this approach in an effort to reduce Zn deficiency [10]. Small-scale farmers usually focus on purchasing fertilizers containing major nutrients nitrogen (N), phosphorus (P) and potassium (K).

### 3.5. Genetic Improvement of Maize for Zn, Provitamin A and Quality Protein

Plant breeding holds great promise for contributing to improvement of the nutritional status of maize and other staple cereal crops across the world [82]. Both national agricultural research (NARS) and international organizations such as the International Maize and Wheat Improvement Centre (CIMMYT), International Institute of Tropical Agriculture (IITA), and HarvestPlus are putting tremendous effort into research and development of biofortified maize cultivars [1,83]. As a result, several varieties of quality protein maize (QPM), enhanced with lysine and tryptophan [84], orange maize which is rich in provitamin A [85] and Zn-enhanced maize [1,86], have been released and commercialized globally. Table 2 shows some of the biofortified varieties released and marketed across the world.

Despite its potential widespread coverage and sustainability, this intervention is facing serious challenges that may need prompt attention. Whilst breeders have succeeded in accumulating high levels of Zn, provitamin A and essential amino acids in maize, all these single nutrient varieties are of less benefit to small-landholder farmers in SSA, whose limited arable land limit crop diversification of these biofortified varieties. Hence, the development of multinutrient maize cultivars, with at least two nutrients among Zn, provitamin A and protein, is an attractive approach to effectively reduce malnutrition challenges in SSA and other maize-based developing countries. It is imperative for breeders to understand the genetic mechanism of QPM, provitamin A and Zn-enhanced maize to facilitate successful integration of these nutritional traits in a single variety.

#### 3.5.1. QPM Genetics and Breeding History

Since the 1960s, scientists have shown great interest in looking for gene mutants that could provide better protein quality in maize grain [94,95]. The discovery of the *opaque-2* mutation in the maize genome was the advent of QPM breeding [14]. After this discovery, many international research organizations invested in QPM breeding. The mutation was targeted to change the *opaque-2* locus from homozygous dominant or heterozygous to homozygous recessive alleles that confer higher tryptophan and lysine content than in normal maize [96]. However, this *opaque-2* mutation came with undesirable phenotypic characteristics of the maize grain [95]. It caused a soft and chalky maize endosperm, which was unacceptable for consumers. In an effort to correct these undesirable effects, further studies focused on the *opaque-2* mutation coupled with genetic manipulation of the *opaque-2* enhancers or modifiers genes [94]. The modification resulted in enhanced transcription of tryptophan and lysine, with consumer preferred hard endosperm characteristics and resistance to ear rots [97].

To retain the QPM genetic background, the development of QPM-based multinutrient maize therefore involves manipulating three distinct genetic systems: (i) the homozygous recessive *opaque-2* locus; (ii) enhancers or modifiers that result in the *opaque-2* gene to confer high lysine and tryptophan and (iii) modifier genes that change the *opaque-2* induced soft endosperm to the desired hard endosperm [20]. In addition to these genetic systems, the QPM-based multinutrient maize should have genetic systems for other nutritional traits as discussed in the following subsections. Multinutrient varieties that have high levels of tryptophan could benefit weaned infants and small children in developing countries that subsist mainly on maize with limited supplementary foods. Unlike provitamin A maize, QPM-based multinutrient maize can be processed into other maize products without much deterioration of its nutritional quality [14]. QPM, in general, has relatively higher niacin or vitamin B3 content and bioavailability due to higher tryptophan and lower leucine content [98].

#### 3.5.2. Provitamin A Maize and Major Carotenoids in Maize Grain

Provitamin A carotenoids are derived from a large class of isoprenoids. There are two main classes of carotenoids, the xanthophylls and carotenes [42]. Xanthophylls are typically yellow, and carotenes are orange pigments. There are several kinds of xanthophylls, including zeaxanthin, lutein, α- and β-cryptoxanthin (βCX), flavoxanthin, neoxanthin and violaxanthin [99]. In this group, only β-cryptoxanthin has vitamin A activity since it contains a single retinyl group [100]. Carotenes are mainly α-, β-, and γ-carotene. All these carotenes possess vitamin A activity in plants, although α- and γ-carotene and the xanthophyll (βCX) produce less vitamin A than β-carotene, which contains two retinyl groups and is enzymatically broken down to retinal or vitamin A [11]. Lycopene has antioxidative properties but has no vitamin A activity [101]. Carotenoids are unsaturated compounds that are highly prone to oxidation, leading to loss of vitamin A activity [78]. The carotenoid degradation mechanisms are highly dependent on the availability of oxygen, light, metals, water and free radicals [41].

Multinutrient maize inclusive of high levels of provitamin A should, therefore, have substantial amounts of dietary carotenoids, in particular β-carotene, α-carotene and β-cryptoxanthin, compared to conventional yellow or white maize. Among cereals, provitamin A maize has been reported to have the highest carotenoid concentration [11]. The most prevalent carotenoids in provitamin A maize are α-carotene, β-carotene, lutein, lycopene, β-cryptoxanthin and zeaxanthin [88]. However, lutein and zeaxanthin are the predominant carotenoids in maize kernels.

Large genetic variation exists for these carotenoids in maize germplasm, making it possible to develop multinutrient cultivars with an added vitamin A nutritional advantage. Provitamin A content in yellow and orange varieties ranges from less than 2 to 25 µg/g [88]. The target for provitamin A content in newly developed maize cultivars has been set by HarvestPlus at 15 µg/g, and several varieties that surpasses this target have been commercialized [18,20,41]. In SSA, maize meal is consumed in large quantities of up to 330 g/person/day, and poor communities consume maize meal several times in a day. Therefore, substituting white maize meal with provitamin A maize creates an opportunity to meet the daily vitamin A requirements. Some studies have reported that provitamin A maize meal can provide more than 50% of the recommended dietary requirement [102]. Therefore, provitamin A maize has great potential to reduce vitamin A deficiency in SSA.

#### 3.5.3. Genetic Basis for High Kernel Zn Content in Maize

Knowledge of the genetic basis of any trait is important in crop improvement. The genetic basis of high grain Zn content in maize was reported to be controlled by many genes, each contributing a small effect to the overall phenotypic expression of this trait [6]. Such polygenic gene action is referred to as quantitative trait loci or QTL. The accumulation of Zn in maize kernels is largely controlled by several factors such as micronutrient availability, uptake by roots, translocation and partitioning to different plant parts, genotypic effects, environmental effects and genotype by environment interaction [1,103]. All these processes are governed by many genes. Very few studies on the genetic mechanism of Zn accumulation in maize kernels have been reported. However, QTL mapping studies reported so far on maize, rice, wheat, barley and Arabidopsis, identified genes related to Zn uptake, transport, phytosiderophore biosynthesis and mineral ion sequestration [1,104,105].

Several studies have suggested the involvement of many QTLs in the accumulation of Zn in maize [1,6,106,107]. Eleven significant QTLs on six chromosomes were identified from a genome wide association study (GWAS) using 923 diverse inbred lines grown in different environments [1]. Different genomic regions were reported [6,107] located on chromosomes 1, 2, 5 and 10 and 1, 2, 6, 7, 9 and 10, respectively. Although some of the identified QTLs from these studies were novel, some were located within many genes involved in Zn uptake and remobilization in plants. Similarly, another study [106] identified 48 candidate genes predicted to be involved in Zn and Fe transport in maize. Several genes were identified from different gene families including the ZIP (zinc-regulated transporter/iron-regulated transporter proteins) family, NRAMP (natural resistance associated macrophage protein) family, YS (yellow stripe) family, CE (cation efflux) family and the ferritin family. All this evidence shows that the accumulation of Zn in maize kernels is complex, with many genes involved.

## 4. Breeding Strategies for Multinutrient Biofortified Maize

**Figure 2:**
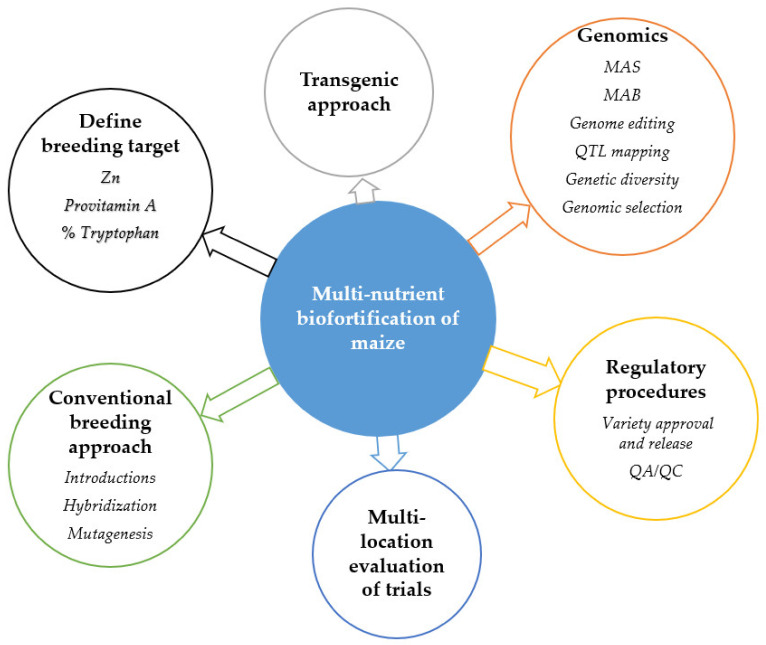
Breeding strategies that can be used for the development of multinutrient maize genotypes. MAS = marker-assisted selection, MAB = marker-assisted backcrossing, QA = quality assurance, QC = Quality control.

The development of multinutrient maize employs a wide range of both conventional and nonconventional breeding strategies. Breeding methods such as introductions, hybridization and mutation breeding, and modern techniques such as marker-assisted and genomic selection, could be used interdependently to increases the rate of genetic gain in breeding for multinutrient maize (Figure 2). Other high-throughput molecular breeding techniques, such as genome editing and genetic engineering, are also useful. Molecular breeding is inclusive of QTL mapping and GWAS, that are widely used to dissect complex traits in maize.

### 4.1. Making Use of the Existing Genetic Variability in Maize Germplasm

The development of multinutrient maize requires the existence of adequate genetic variability for targeted nutrient concentration [108]. This enables efficient selection of the best cultivars with the desired traits. Interestingly, maize has considerable genetic variability for several nutritional, yield and morpho-physiological traits [3,11,37]. The genetic differences are attributed to different alleles of a particular gene that frequently occur in a diverse population [109]. Some of the desired alleles for nutritional traits are prevalent in landraces, wild relatives and improved germplasm cultivated across the world. Variability in nutritional traits such as phlobaphenes (red maize), anthocyanins (blue, black and purple maize), carotenoids (orange and yellow maize) and minerals (Zn and Fe), exists in landraces maintained at the gene bank of CIMMYT [110], and breeders from both national and international breeding programs can take advantage of this genetic resource.

Several studies have been conducted to evaluate genetic variability of Zn [3,108,111] and provitamin A concentration in maize endosperm [5,112]. Studies evaluating the protein content, essential amino acids content of lysine and tryptophan and quality index (% tryptophan/protein content) indicate that even in QPM germplasm, large differences of these nutritional attributes exist [84]. Lysine content in QPM was reported to vary from about 3.3 to 4.0 g per 100 g of endosperm protein, which is more than twice that of normal maize endosperm [14,28]. Despite this variation, QPM contains about 55% more tryptophan and 30% more lysine than normal maize, although this varies [7,113]. Significant variation of Zn content in normal (nonbiofortified) tropical maize inbred lines was observed [108], ranging from 17.5 to 42 mg/kg of Zn. Similarly, kernel Zn content was evaluated [114] using a core population of 30 diverse maize genotypes for consecutive rainy seasons in 2006, 2007 and 2008, and considerable genetic variability of 15.14 to 52.95 mg/kg was observed.

Apart from these findings, several studies have reported higher Zn content in QPM germplasm than maize from other nutritional profiles [1,103,115]. This is encouraging and can facilitate the development of multinutrient cultivars enhanced with Zn and protein quality. Variability of 143 to 278 µg/g of the total carotenoids was reported [5] after evaluating 22 tropical maize inbred lines from different genetic backgrounds. Variability of provitamin A carotenoids content in separate forms, such as β-carotene, α-carotene and β-cryptoxanthin, has also been reported. Although β-carotene has the highest provitamin A activity, it is present in a relatively low concentration of 0.5–2.5 µg/g in most orange or yellow maize grown across the world [88,112]. To date, CIMMYT has hundreds of provitamin A genotypes with varying levels of provitamin A from less than 2 µg/g to >25 µg/g [20,116,117]. However, very few studies have reported on mineral concentrations in provitamin A germplasm. Therefore, screening of the available provitamin A germplasm for Zn and other minerals can be a good starting point for breeders in the pursuit of multinutrient biofortification.

Considering the available genetic variability for Zn and provitamin A content in maize, and the EAR of Zn and provitamin A, HarvestPlus, in collaboration with plant breeders, scientists and food processors, has set a target of 33 and 15 µg/g of Zn and provitamin A content in maize kernels, respectively. The baseline content for Zn is 20 µg/g and considering the wide variability of Zn content in maize an increase of 13 µg/g is feasible [1,20]. Despite all these breeding efforts, the availability of Zn in maize endosperm is highly dependent on agronomic management such as application of Zn containing fertilizers, since most soils in SSA are Zn-deficit. It is, therefore, advisable to know the Zn content of the soils where genotypes evaluated for Zn content in kernels are planted. Lastly, the knowledge of extent of genetic variability for the targeted nutritional traits in locally adapted germplasm is important to breeders in pursuit of multinutrient maize biofortification. Genotypes with high nutrient content could be used in crosses, genetic studies and for developing gene pools and mapping populations [117].

### 4.2. Germplasm Introductions and Testing for Stability in Local Environments

Only a few countries have sufficient plant genetic resources to fulfil their food requirements [118]. Improved germplasm in modern days is, to a lesser extent, associated with the centers of diversity, but is sourced from national and international gene banks such as the CIMMYT gene bank. Such germplasm may be in the form of landraces or improved genotypes that can be introduced to other countries for cultivation. Germplasm introduction is among breeding strategies that have been used by breeders for many years. Currently, CIMMYT and IITA are developing genotypes with a wide range of attributes, including tolerance to biotic and abiotic stress, high yield potential and nutritional quality, that are accessible to plant breeders across the world [37]. As a result, provitamin A, Zn and QPM donor inbred lines can be acquired from CIMMYT and IITA and introduced to different parts of the world. Recently, CIMMYT has embarked on extensive screening of normal and biofortified germplasm such as QPM for mineral content [20,109,115]. Such initiatives may result in some genotypes with one or more nutrients being identified. Introduced germplasm, however, requires extensive evaluation for stability across a wide range of growing environments in the respective countries.

### 4.3. Exploiting Heterosis through Hybridization

Multinutrient biofortified maize cultivars can be developed through hybridization. Although different population improvement procedures exist, the development of multinutrient cultivars through hybridization is highly influenced by several factors, including availability of resources, genetic variability, breeders’ expertise and high-throughput phenotyping tools. Using this breeding method, breeders can develop either multinutrient hybrids or improved open pollinated varieties (OPVs) [117]. Nutritionally-dense improved OPVs are developed as multiple line synthetics that harbor several desirable alleles, such as resistance to biotic and abiotic stress factors. Hence, developing multinutrient OPVs in SSA is attractive, especially in West Africa where OPVs occupy more than 60% of the formal seed sector [119]. Unlike hybrids, resource-poor farmers can recycle improved multinutrient OPVs for about three to four years without much yield loss. Moreover, certified seed for OPVs is relatively cheap compared to hybrids, which ultimately improves accessibility of improved seed by poor target communities. Despite all these advantages, multinutrient OPV cultivars may yield up to 30% less than hybrids [120].

Prevalence of additive gene action for traits such as Zn, provitamin A and quality protein, facilitates the development of improved multinutrient OPVs through recurrent selection. This can be achieved by crossing an improved OPV with a trait donor, followed by backcrossing with the recurrent parent to restore desirable traits in the original cultivar. Therefore, intrapopulation recurrent selection and pedigree selection are useful breeding methods that can be used for developing multinutrient maize OPV. The main disadvantage of recurrent selection is that some of the desirable traits come as favorable alleles in haplotype blocks and, therefore, some of these alleles can be lost during the integration of targeted traits [121]. Maintenance of multinutrient OPVs without considerable yield loss is highly dependent on the degree of isolation from foreign pollen, causing seed admixture with other varieties. Removal of off-types is, therefore, critical in maintaining the genetic purity of multinutrient OPVs. To date, numerous QPM and provitamin A OPVs have been released in more than 30 countries across the world. For instance, Obantanpa is a popular open-pollinated QPM widely grown in east and west parts of Africa [122]. The nutritional value of such popular OPVs can be further improved with other nutrients using interpopulation recurrent selection procedures.

Hybrid development is another breeding approach for developing multinutrient maize cultivars. Whilst other breeding methods are useful, the genetic improvement of maize across the world remains centered on hybrid development [123]. Multinutrient hybrids are developed by crossing two or more inbred lines (with target nutrients) from diverse populations to exploit heterosis or hybrid vigor. In general, hybrid cultivars dominate the formal seed sector in the southern parts of Africa, where more than 80% of the cultivars on the market are hybrids [124]. For this region, it could be of great benefit to farmers if multinutrient hybrid cultivars are developed as single, three-way and double crosses. Single crosses have high yield potential, but high cost of seed production is a major limitation due to low yield potential of inbred lines used as females. Hence, three-way hybrids may occupy a larger market segment in this region than single crosses.

The success of multinutrient hybrid maize breeding is highly dependent on the level of heritability of targeted nutritional traits, mode of gene action and general and specific combining ability (GCA and SCA) of the parental inbred lines. For kernel Zn content, high narrow-sense heritability of more than 72% was reported in QTL mapping studies [1,40]. Medium to high heritability for provitamin A content was also reported [112]. High broad-sense heritability of about 85% for kernel Zn content was reported in QPM genotypes [109]. In addition, several studies were conducted to evaluate the GCA and SCA estimates of Zn, provitamin A and QPM inbred lines in an effort to develop hybrids with high yield potentials. Higher GCA effects than SCA were reported [125] using testcross hybrids from a diallel cross of inbred lines contrasting for kernel Zn concentration. Another study [84] found significant GCA and SCA for quality index (% tryptophan/protein content) from a diallel cross of QPM inbred lines, although GCA effects were more important than SCA effects. Significant GCA effects for provitamin A content, and weak and nonsignificant SCA effects in elite provitamin A lines, was also found [116]. Prevalence of GCA effects on multilocational trials shows that additive effects are more important than nonadditive or epistatic gene action for these quality traits.

### 4.4. Marker-Assisted Breeding

Marker assisted selection (MAS) or breeding could bring a high level of success in developing multinutrient maize. MAS is an indirect selection process where selection of the desired trait is done based on a specific marker, which can be morphological, biochemical or DNA/RNA markers (known as molecular markers). This modern breeding tool has been used in developing provitamin A cultivars, where tropical and temperate germplasm with high carotenoid content was selected based on the presence of reduced-function alleles of the lycopene epsilon-cyclase (*LcyE*) [42] and β-carotene hydroxylase 1 (*CrtRB1*) [116,126]. The presence of molecular markers for genes, including phytoene synthase 1 (*PSY1*), facilitates development of multinutrient maize on a provitamin A background, by tracking the presence of these favorable alleles that confer increased β-carotene content. Identification of these favorable alleles using PCR-based markers, coupled with high-throughput phenotyping tools, such as high-performance liquid chromatography (HPLC), is crucial in accelerating genetic gains in developing multinutrient maize in a provitamin A background.

MAS can also be used to confirm the presence of the *opaque-2* locus in QPM donors to be used for QPM-based multinutrient maize. Marker-assisted backcrossing (MAB) could be useful in ensuring successful introgression of the *opaque-2* loci and other genetic systems controlling amino acid content [127]. Successful MAB of favorable alleles of *CrtRB1* from a provitamin A donor (HP321-1) to two QPM inbred lines (CML161 and CML171) was reported to improve both provitamin A and protein quality [128]. High background recovery rates of 89.9% and 92.1% in the BC2F2 generation were reported for the QPM recurrent parents, respectively, and provitamin A content improved from 1.60 to 5.25 µg/g (CML161) and 1.80 to 8.14 µg/g (CML171). Similarly, multinutrient maize hybrids were developed by marker-assisted stacking of *CrtRB1*, *LcyE* and *opaque-2* loci in the QPM genetic background, resulting in cultivars with high levels of provitamin A, lysine and tryptophan content [129].

The use of DNA-based markers such as intersimple sequence repeat (ISSR), simple sequence repeat (SSR), random amplified polymorphic DNA and single nucleotide polymorphism (SNPs), can also be used for estimating genetic distance between inbred lines, studying population structure and classifying multinutrient germplasm into heterotic groups [130]. The use of ISSR diagnostic markers in a cross involving QPM by non-QPM lines (i.e., Zn, normal or provitamin A), showed the possibility of developing molecular breeding programs for multinutrient maize with a QPM genetic background [131]. In Uganda, three SSR markers for the *opaque-2* locus have been identified as *phi057*, *phi112* and *umc1066*. Among these, the *phi057* and *phi112* were reported to be highly polymorphic and, therefore, can be used for introgression of the *opaque-2* gene in other biofortified germplasm with a non-QPM background [131]. The presence of these polymorphic markers was associated with high levels of tryptophan content in maize kernels, which is quite encouraging to breeders. Highly polymorphic markers such as SNPs are useful in genomic selection, QTL mapping and GWAS to identify genomic regions influencing high levels of nutritional traits. For instance, more than 20 SNPs were identified that have direct influence on the accumulation of Zn content in maize kernels [1].

### 4.5. Mutation Breeding

Mutation breeding could be useful in developing multinutrient maize genotypes. This strategy has been used for many years to create genetic variability in both quantitative and qualitative traits. Different mutagens, including X-rays, gamma-rays and chemical mutagens, such as ethyl methyl sulfonate (EMS), have been widely used in different crops to induce random changes in DNA. Exposing maize seed to different levels of mutagen doses can help to identify optimal doses that can cause significant point mutations without causing much of the undesirable characteristics. M1 generation kernels are advanced and desirable phenotypic effects can be identified, depending on the dominant or recessive nature of alleles. Although there is little evidence on the development of multinutrient maize genotypes using this breeding method, few studies have reported significant changes of yellow to orange maize when EMS was used on M1 yellow maize segregating populations [37,42]. Mutation induction, however, could cause undesirable traits linked to the desired trait. Such traits include albinism, increased kernel abortion and increased susceptibility to biotic and abiotic stress factors [132].

Mapping studies to identify loci with favorable alleles in mutation breeding can be done using Targeting Induced Local Lesions in Genomes (TILLING). This high-throughput technique can identify the extent of genetic variability for a particular trait in locally available germplasm. TILLING is a reverse genetics tool based on conformational electrophoresis for identifying point mutations in plant populations [133]. Multinutrient varieties developed through TILLING are not subject to regulatory requirements for approval as encountered with transgenic varieties.

### 4.6. Use of Transgenics in Developing Multinutrient Maize

Genetic engineering of maize to develop nutritionally superior maize genotypes could be an efficient biotechnological breeding approach that could reduce some of the hurdles faced by breeders in developing multinutrient maize. Transgenic techniques have been advocated as breeding tools for some traits, due to limited success of conventional breeding methods to incorporate desired traits [42]. Introgression of desired alleles in a wide range of germplasm with different nutritional attributes may be successful after many years of crosses and QTL mapping studies [37]. Several methods have been proposed to introduce transgenes in the maize genome, including *Agrobacterium tumefaciens*-mediated transformation, microparticle bombardment, and whiskers-mediated transformation [134]. Transgenic maize cultivars with insect and herbicide resistance are widely grown in both North and Latin America. Male sterile inbred lines have also been developed to eliminate detasselling costs in hybrid development.

Multinutrient maize in a provitamin A genetic background could be developed through manipulation of the carotenoid biosynthetic pathway [122]. Several studies have reported successful development of biofortified transgenic maize varieties. For instance, successful integration of transgene *sb401* encoding a lysine-rich protein into the maize genome was observed to increase lysine and total protein content in the transgenic QPM [135]. The development of Golden Rice is a good example of biofortified crops developed through genetic engineering [136]. A similar transgenic approach has been used to improve normal white or yellow Zn enhanced and QPM genotypes, with high provitamin A content, through overexpression of the bacterial genes *crtB* and *crtL*, resulting in a 34-fold increase of total carotenoids in the maize endosperm [137]. This is encouraging, as this shows some breeding efforts to stack several nutritional attributes in maize cultivars as a way of improving its poor nutritional status. Although several transgenic crops have been developed worldwide, and many studies have shown that GM crops are safe for both human consumption and the environment, continued skepticism around transgenic crops is likely to affect full adoption of this technology in some countries. Transformation of crops with foreign genes has attracted an unresolved debate around biosafety issues, regulatory requirements and restrictive government policies.

### 4.7. Genome Editing

Genome editing is a powerful biotechnological tool that can be used to stack nutrients in maize. Unlike in GM crops, where transgenes are involved, genome editing techniques insert edited genes of interest into specific genomic regions and the procedure mimics the natural hybridization process [138]. Techniques such as Zinc Finger Nucleases (ZFN) and Transcription Activator-Like Effector Nucleases (TALENs) have been widely used for genome editing. Recent development on genome editing tools, such as Clustered Regulatory Interspaced Palindromic Repeats (CRISPR), enables precise modifications in the genome with high reproducibility and avoid cellular toxicity [37].

Gene editing has been used to improve traits for several crops. For instance, yield and stress tolerance in rice [139], β-carotene content in Cavendish banana [140], reduced phytic acid content in maize kernels, powdery mildew resistant in wheat and drought tolerant maize [141]. Therefore, multinutrient maize can be developed using such cutting-edge technologies that have great potential to receive a wider public acceptance compared to transgenic crops.

## 5. Major Challenges in Developing Multinutrient Maize

The development of multinutrient maize with increased concentrations of both macro and micronutrients creates great opportunity to alleviate malnutrition in SSA. However, factors that may contribute to relatively low adoption of multinutrient cultivars should be considered. Reflecting on challenges previously experienced in adoption of these single nutrient varieties, provides a guideline to researchers in pursuit of stacking these nutrients in maize cultivars.

### 5.1. Acceptance of Multinutrient Maize in a QPM Background

Multinutrient maize cultivars developed in a QPM background may face similar adoption challenges to those faced by the conventional QPM varieties [142]. Firstly, the genetic nature of QPM in QPM-based multinutrient cultivars may have a negative effect on its adoption by farmers. Because the *opaque-2* locus that comes with the QPM genetic background is homozygous and recessive, extra care has to be taken in seed production to reduce xenia effects from non-QPM cultivars [95,113]. Xenia effects can also negatively affect the nutritional composition of QPM-based multinutrient maize and, therefore, growing it in isolation, either by time or by distance, is of paramount importance. Isolation distances can vary from one country to another, but in general, isolation distances of 400 m are commonly used. Where farmers cannot afford the stipulated isolation distances due to smaller pieces of arable land, several border rows can be planted to protect the multinutrient cultivar from potential contaminants. Furthermore, multinutrient genotypes developed from white colored Zn-enhanced and QPM maize cannot be easily distinguished from the conventional maize, which ultimately affects its full adoption by consumers [97]. However, with provision of adequate farmer and consumer education, QPM-based multinutrient maize could be fully adopted.

### 5.2. Acceptance of Multinutrient Maize in a Provitamin A Background

Knowledge gaps, cultural beliefs and behavioral patterns are still creating barriers to production and consumption of any orange-colored maize in developing countries [87,143]. However, some studies reported that the provision of adequate nutritional information and constantly educating the public of the nutritional benefits of provitamin A maize can improve its adoption [28,144,145]. Despite all these efforts, the orange color of multinutrient maize developed from a provitamin A genetic background, may affect its acceptance since the majority of consumers in SSA prefer white maize to orange or any yellow-colored maize [41,42]. This negative perception started during the colonial period, when white-dent maize was first introduced in Africa, and since then consumers became accustomed to white maize [144]. From that time, any orange or yellow colored maize was perceived as unsuitable for human consumption and was used as livestock feed [87,146]. In addition, provitamin A maize has been reported to have a different flavor and aroma compared to white maize [147]. Women have been reported to experience challenges in feeding children who are not familiar with the orange color [147]. This nonpreference behavior may have been caused by lack of nutritional information and advocacy to the targeted people.

It is encouraging that the orange-fleshed sweet potato (OFSP) was accepted in Mozambique and all its neighboring countries due to proper consumer education. Lessons learnt from the acceptance of orange-sweet potato raises optimism for the acceptance of multinutrient maize rich in provitamin A [41]. Current breeding efforts at CIMMYT are aimed at developing high yielding and stress tolerant provitamin A cultivars as a way of increasing their adoption. Hence, orange-colored multinutrient maize is likely to be embraced if it is readily available in shops and has similar agronomic, culinary and sensory characteristics as normal maize [143].

Pricing of the multinutrient orange colored maize should be done in a strategic manner to ensure both affordability by poor consumers and acceptance by rich consumers who might associate lower prices with low quality foods [147]. Since provitamin A carotenoids are highly oxidative, proper storage of provitamin A cultivars is required to minimize carotenoid losses due to degradation [8,88]. However, a high rate of degradation has been reported in milled maize flour stored in translucent packaging compared to maize grain [89]. This is because milled flour has a high surface area as a result of milling and, therefore, has increased exposure to oxygen, light and other pro-oxidant environments. It was reported [148] that the rate of carotenoid degradation is less in refined maize meal than for whole grain meal because the high fat content in the germ causes rancidity, and this produces undesirable odors and flavors. To address these storage challenges, scientists are promoting the use of Purdue Improved Crop Storage (PICS) bags in African countries for post-harvest storage of provitamin A maize [89]. This is achieved through a decrease in oxygen and an increase in carbon dioxide, resulting in increased carotenoid stability and retention in maize to over six months as compared to storage in polypropylene bags [41]. In addition to PICS bags, there is still need for research on the best and affordable on-farm storage facilities for multinutrient maize on provitamin A background.

### 5.3. Acceptance of Multinutrient Maize on Zn Genetic Background

The development of maize cultivars with increased micromineral content is currently being advocated by HarvestPlus. Previously, research and development has been focusing on development of provitamin A and QPM cultivars. Hence, Zn-enhanced maize is quite new to the majority of people in SSA. This implies that research organizations should invest in promoting Zn biofortified maize. Although the rate of acceptance of newly biofortified maize may differ across different countries and regions, gradual changes in consumer behavioral patterns may be expected through consumer education [149].

Access to seed by farmers also influences adoption of Zn biofortified maize [110]. In fact, the provision of subsidized seed can facilitate quick adoption. For instance, provision of plant material for OFSP in Rwanda led to full adoption of provitamin A sweet potato [149]. A similar approach has been implemented by HarvestPlus to improve seed availability of Zn and Fe-enhanced beans, and such an approach could work for the newly developed Zn-rich multinutrient maize. Other platforms to promote Zn-enhanced maize include media, agricultural shows, seed fairs and field days [113]. The involvement of extension workers is crucial, since they are drivers of new technology such as the promotion of Zn-biofortified maize. Another potential challenge of Zn-enhanced maize in general, is the need to apply Zn fertilizers in order to reach its full potential. This is important in SSA, since most arable soils have low Zn concentration and, therefore, farmers may incur extra Zn-containing fertilizer costs [10].

### 5.4. Low Yield Potential of Biofortified Maize Cultivars

Unfortunately, all biofortified maize are perceived as low yielding compared to normal maize. Some studies report that the accumulation of nutrients in maize kernels through biofortification carries a yield penalty [9]. However, several studies indicated that improved nutritional quality does not negatively affect the cultivar yield potential [84]. In fact, some biofortified varieties such as QPM have been reported to outperform some of the conventional maize varieties [40,132]. Correlation studies on provitamin A maize showed that grain yield is not significantly correlated with carotenoid content [11]. Some of the recently developed provitamin A, Zn and QPM hybrids have yield potential of more than 8 ton/ha, showing good potential for developing multinutrient maize [1,109,132]. Although these studies show encouraging results, there is a need to investigate genotype by environment interaction of biofortified maize to ensure that cultivars are recommended for suitable growing environments. The low yields observed in some cases could be a result of a narrow genetic base of biofortified germplasm. In that case, classifying biofortified inbred lines into different heterotic groups can help in exploiting heterosis in biofortified maize. Biofortified germplasm with resistance/tolerance to biotic and abiotic stresses, such as maize streak virus, grey leaf spot, *Turcicum* leaf blight, fall armyworm, drought, heat and low nitrogen, can also help to increase yields.

### 5.5. Quality Assurance for Multinutrient Maize

Multinutrient maize requires efficient monitoring systems to ensure that the original nutritional quality is maintained. One of the strategies to ensure production of high quality multinutrient maize is the use of isolation distances to protect the crop from contamination. Isolation by time can also be used by farmers, where planting is done on different times to ensure that flowering periods of the varieties do not coincide. Quality analysis should also be performed at all stages during cultivar development, production and storage to ensure that nutritional quality is guaranteed [41,96]. Quality control is also critical during inbred line development and maintenance. Multinutrient maize involving provitamin A germplasm requires sophisticated quality analysis tools such as HPLC. Zn content in kernels is quantified using advanced spectrophotometers such as near-infrared reflectance, X-ray fluorescence and inductively coupled plasma (ICP) spectrophotometers [38,40]. All this equipment requires significant financial resources, which most food processors and research organizations may not be able to afford. Wet analytical procedures for Zn, carotenoids, and quality protein requires expensive chemical reagents, which may also need substantial investments [40].

### 5.6. Policy Regulations

National policy regulations have an impact on the adoption of multinutrient maize. Without support from governments, the development and production of multinutrient maize can be a challenge. Enabling policy environment in this context includes supporting the whole value chain to develop and deploy multinutrient cultivars. Strengthening of seed systems to ensure easy access of subsidized biofortified seed and inputs is also important. However, cultivars developed using genetic engineering are prohibited in some countries in SSA. Such restrictive government policies limit the World Health Organization to reach its sustainable development goals for 2030 to end hunger in all its forms [51]. Therefore, the involvement of policy makers in awareness and educational campaigns advocating policy change to permit either genome-edited or transgenic multinutrient maize could help in combating malnutrition in high-risk societies.

## 6. Conclusions

The development of multinutrient maize with Zn, provitamin A and QPM can reduce malnutrition in developing countries. Multinutrient maize cultivars can be developed using the available genetic variation for these traits, and integration of both conventional and modern high-throughput breeding methods. Such breeding methods include conventional pedigree selection, marker-assisted breeding, genetic engineering and genomic selection. Breeders can make use of the gene bank collections preserved at CIMMYT to acquire maize nutrient donors that can be accessed by both national and international breeding programs. In addition, screening of the existing germplasm for carotenoids, Zn and tryptophan content is important and can identify nutritious maize genotypes that are highly adapted to local growing conditions. Promotion of multinutrient maize cultivars should be done to facilitate quick adoption by farmers and consumers. Availability of affordable multinutrient maize in shops, and provision of seed subsidies, can facilitate its quick adoption. Apart from the nutritional attributes, multinutrient maize cultivars should have good agronomic traits such as tolerance to biotic and abiotic stresses. Quality assurance at all breeding and seed production stages should be monitored to ensure the nutritive value of multinutrient maize.

## Figures and Tables

**Table 1:** Ranking of the ten leading health risk factor causes of Disability Adjusted Life Years (DALYs) in low-income countries [32].

Risk Factor	DALYs (millions)	Risk Factor	DALYs (millions)
World		Low-income countries	
Underweight	91	Underweight	82
Unsafe sex	70	Unsafe water	53
Alcohol	69	Unsafe sex	52
Unsafe water	64	Suboptimal breastfeeding	34
Blood pressure	57	Indoor smoking	33
Tobacco use	57	Vitamin A deficiency	20
Suboptimal breastfeeding	44	Blood pressure	18
High blood glucose	41	Alcohol	18
Indoor smoking	41	High blood glucose	16
Obesity	36	Zinc deficiency	14

**Table 2:** List of some of the provitamin A, Zn and QPM biofortified maize varieties released in different countries across the world.

Variety	Target Trait	Target Countries	Year of Release	Reference
BIO-MZN01	Zinc	Columbia	2018	[1]
ICTA HB-15	Zinc	Guatemala	2018	[40]
ICTA B-15	Zinc	Guatemala	2018	[40]
GV665A	Provitamin A	Zambia	2012	[87]
GV662A	Provitamin A	Zambia	2012	[88]
Abontem	Provitamin A	Ghana	2012	[89]
MH39A, MH40A	Provitamin A	Malawi	2016	[89]
ZS242A	Provitamin A	Zimbabwe	2015	[86]
RAHA02	Provitamin A	Rwanda	2017	[89]
HQPM-5	QPM	India	2007	[46]
Obatanpa	QPM	Ghana	1992	[90]
ZS261	QPM	Zimbabwe	2006	[91]
BHQP542	QPM	Ethiopia	2001	[92]
Q623	QPM	South Africa	2014	[93]
Yanrui-1	QPM	China	2010	[93]

ProA = provitamin A, QPM = quality protein maize.

## Data Availability

No data related with review.
